# Data from multimodal functions based on an array of photovoltaic modules and an approximation with artificial neural networks as a scenario for testing optimization algorithms

**DOI:** 10.1016/j.dib.2019.104669

**Published:** 2019-10-16

**Authors:** Carlos Robles-Algarín, Diego Restrepo-Leal, Adalberto Ospino Castro

**Affiliations:** a Universidad del Magdalena, Facultad de Ingeniería, Carrera 32 No 22 – 08, Santa Marta, Colombia; b Universidad de la Costa, Facultad de Ingeniería, Calle 58 No 55-66, Barranquilla, Colombia

**Keywords:** Artificial neural networks, Multimodal functions, Optimization algorithms, Partial shading, Photovoltaic modules

## Abstract

This paper presents the data of multimodal functions that emulate the performance of an array of five photovoltaic modules under partial shading conditions. These functions were obtained through mathematical modeling and represent the P–V curves of a photovoltaic module with several local maximums and a global maximum. In addition, data from a feedforward neural network are shown, which represent an approximation of the multimodal functions that were obtained with mathematical modeling. The modeling of multimodal functions, the architecture of the neural network and the use of the data were discussed in our previous work entitled “Search for Global Maxima in Multimodal Functions by Applying Numerical Optimization Algorithms: A Comparison Between Golden Section and Simulated Annealing” [1]. Data were obtained through simulations in a C code, which were exported to DAT files and subsequently organized into four Excel tables. Each table shows the voltage and power data for the five modules of the photovoltaic array, for multimodal functions and for the approximation of the multimodal functions implemented by the artificial neural network. In this way, a dataset that can be used to evaluate the performance of optimization algorithms and system identification techniques applied in multimodal functions was obtained.

**Table:** Specifications Table

Subject	Computer Science
Specific subject area	Artificial Intelligence, Photovoltaic Energy
Type of data	Table, Figures
How data were acquired	Numerical simulation based on a code written in C language, for an array of five 65 W photovoltaic modules. The MATLAB/Simulink Neural Network Toolbox was used for training the artificial neural networks. The code developed is attached in the supplementary material.
Data format	Raw, Filtered and analyzed
Parameters for data collection	The data obtained from the array of the five photovoltaic modules were normalized in order to facilitate the training of the artificial neural network.
Description of data collection	The data were obtained from a mathematical model that represents the behavior of a photovoltaic (PV) module. The model has as inputs the solar irradiance and the operating temperature. The outputs correspond to the voltage and power of the PV module. With this mathematical model, an array of five modules was designed to obtain multimodal functions. For irradiance, input values between 10 and 1000 W/m^2^ were generated, while for temperature, values between 5 and 150 °C were used. To each PV module of the array, different values were applied to the inputs, in order to represent the partial shading conditions. The output data for power and voltage were exported in DAT files. In this way, the data for four multimodal functions were obtained. Finally, an artificial neural network was designed to obtain the output data of the power and voltage of four approximation functions representing the four multimodal functions.
Data source location	Universidad del Magdalena, Santa Marta, Colombia.
Data accessibility	Data are provided in supplementary materials with this manuscript.
Related research article	Guillot, J.; Restrepo-Leal, D.; Robles-Algarín, C.; Oliveros, I. Search for Global Maxima in Multimodal Functions by Applying Numerical Optimization Algorithms: A Comparison between Golden Section and Simulated Annealing. Computation 2019, 7 (3), 43, DOI: https://doi.org/10.3390/computation7030043.

**Table:** 

**Value of the Data** • The data presented in this paper can save time for other researchers who need to apply this data to evaluate the performance of different types of optimization algorithms applied in multimodal functions. • Using these data, researchers can evaluate the performance of intelligent control algorithms applied to the maximum power point controllers, in scenarios where photovoltaic modules are exposed to partial shading conditions. • These data are useful for applying system identification techniques, in order to obtain mathematical models that represent multimodal functions with multiple local maximums and a global maximum. • The dataset provided can be used to analyze the operation of PV modules that are exposed to dynamic values of solar irradiance and operating temperature, which emulates the partial shading effect that occurs in PV systems.

## 1 Data

The data of the multimodal functions presented in this article were obtained through simulation of an array of five 65 W photovoltaic modules type YL65P-17b.

**Fig. 1:**
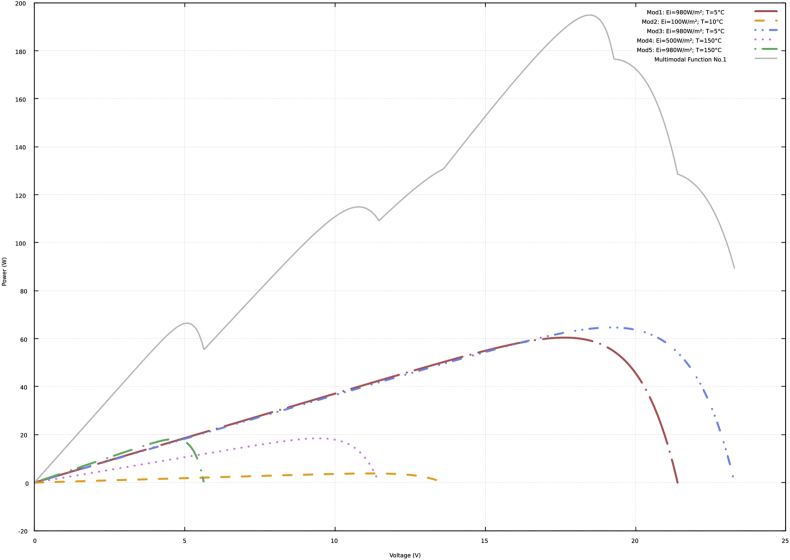
P–V curves and multimodal function No. 1.

**Fig. 2:**
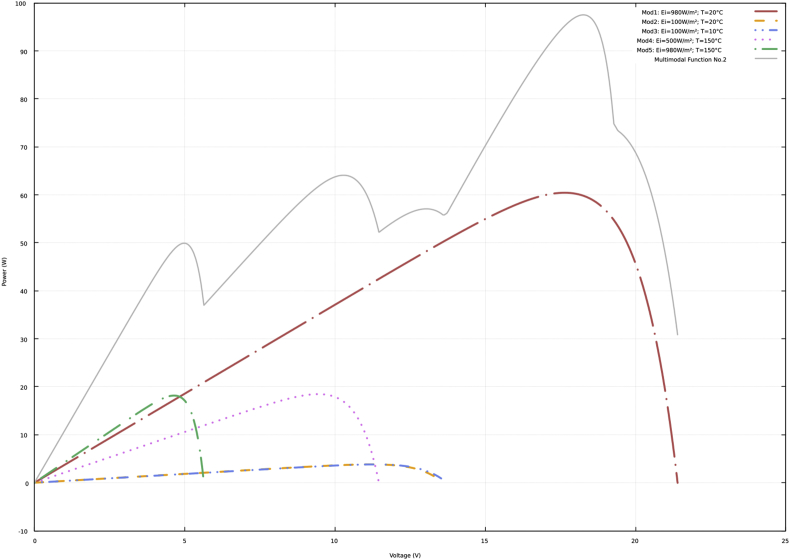
P–V curves and multimodal function No. 2.

**Fig. 3:**
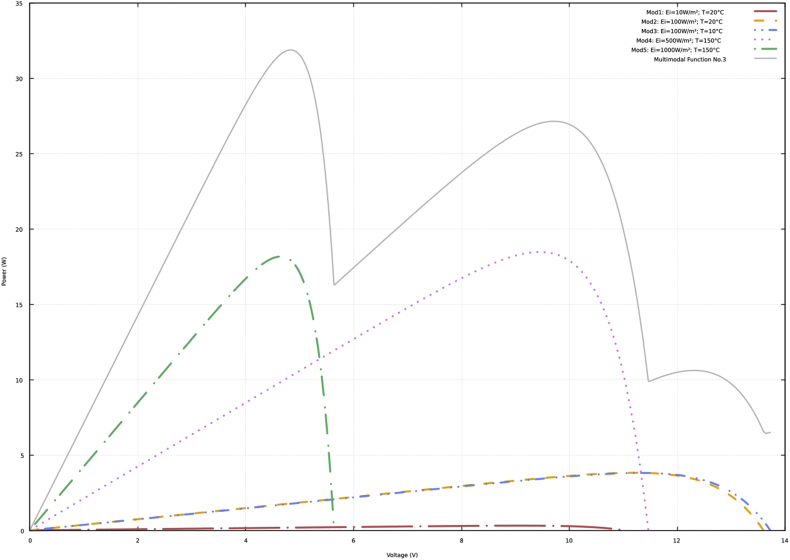
P–V curves and multimodal function No. 3.

**Fig. 4:**
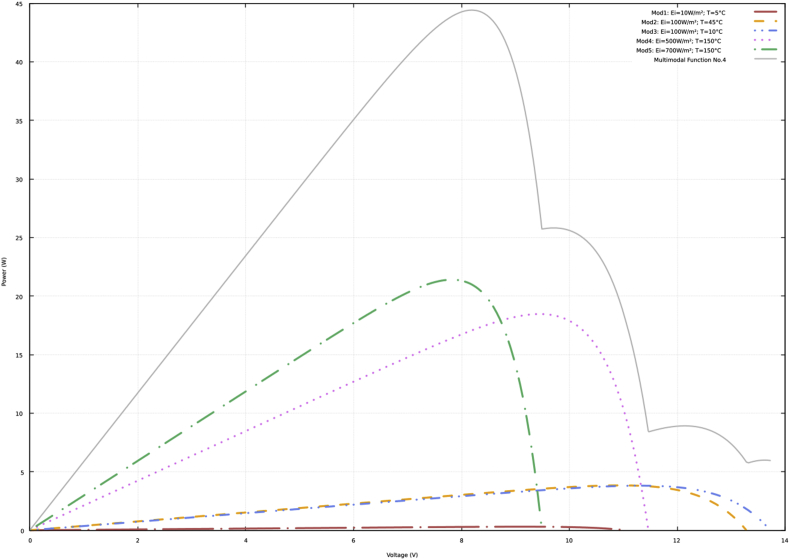
P–V curves and multimodal function No. 4.

In Fig. 1, Fig. 2, Fig. 3, Fig. 4, the four multimodal functions are shown, which differ in the number and location of the maximums. In each of the figures there are six curves, of which five correspond to the P–V curves of each of the photovoltaic modules of the array. Each of these curves was generated using different values of solar irradiance and operating temperature for each PV module in the array. The other curve is the multimodal function obtained with the sum of the previous ones, which also represents a P–V curve that emulates partial shading conditions.

Tables 1–4 of the supplementary material show the data of the P–V curves (power in watts and voltage in volts) according to the previous description and the information presented in Fig. 1, Fig. 2, Fig. 3, Fig. 4. In addition, the data obtained with the neural network to approximate the four multimodal functions are presented.

The data presented in the four tables of the supplementary material have the same structure. Initially, the power and voltage data of each of five PV modules are presented. Subsequently, there are the power and voltage data of the multimodal function. Finally, there are the power and voltage data obtained with the neural network.

## 2 Experimental design, materials, and methods

To represent the multimodal functions, the mathematical model shown in equation (1) was used, which shows the current-voltage ratio (I–V) of a PV module [2].

(1) $$I\left(V\right)=\frac{I_{x}}{1-e^{\left(\frac{-1}{b}\right)}}\left[1-e^{\left(\frac{V}{bV_{x}}-\frac{1}{b}\right)}\right]\;$$

where *V*_*x*_ and *I*_*x*_ represent the open circuit voltage and the short-circuit current of the PV module for dynamic values of solar irradiance and operating temperature.

(2) $$V_{x}=s\frac{E_{i}}{E_{iN}}TC_{v}\left(T-T_{N}\right)+sV_{max}-s\left(V_{max}-V_{min}\right)e^{\left(\frac{E_{i}}{E_{iN}}ln\left(\frac{V_{max}-V_{oc}}{V_{max}-V_{min}}\right)\right)}\;$$

(3) $$I_{x}=p\frac{E_{i}}{E_{iN}}\left[I_{sc}+TC_{i}\left(T-T_{N}\right)\right]\;$$

where *s* and *p* represent the number of PV modules in series and parallel. The parameters used for modeling are shown in Table 1.

**Table 1:** Specifications of the photovoltaic module.

Parameter	Description	Value
V_MPP_	Voltage at maximum point	17.5 V
I_MPP_	Current at the maximum point	3.71 A
I_sc_	Short-circuit current	4 A
V_oc_	Open circuit voltage	21.7 V
T_cv_	Temperature coefficient of voltage	−0.0802 V/°C
T_ci_	Temperature coefficient of current	0.0024 A/°C
E_i_	Variable solar irradiance	Variable (W/m^2^)
E_iN_	Constant solar irradiance value	1000 W/m^2^
T	Variable operating temperature	Variable (°C)
T_N_	Constant temperature value	25 °C
V_MAX_	Maximum voltage of the PV module	22.35 V
V_MIN_	Minimum voltage of the PV module	18.44 V

With the mathematical model described by Equations (1), (2), (3) and with the parameters established in Table 1, a C code was performed to model the performance of a PV module for dynamic values of solar irradiance and operating temperature.

Each multimodal function was obtained from an array of five PV modules. Each module was configured to operate with different values of solar irradiance and operating temperature, obtaining five series of data which were named as follows in the supplementary file: Power of the PV Module No.1, Power of the PV Module No.2, Power of the PV Module No.3, Power of the PV Module No.4 and Power of the PV Module No.5. Each of these data series corresponds to the ideal curve that characterizes the P–V curve of a PV module.

By varying the irradiance and temperature, the P–V curve is affected on the x and y axes. Consequently, the number of samples between the data sets are not uniform in any of the axes, some contain more samples than others. For this reason, to obtain the multimodal functions, all the y-axis values are added, while for the x-axis, the voltage data with the largest number of samples must be selected. Thus, multimodal functions are made up of the sum of all the values of the y-axis and the set of the greatest amount of data on the x-axis.

With the procedure described above, five multimodal functions were obtained representing the P–V curves of a PV module with multiple local maximums and a global maximum. This situation shows the performance of PV modules in partial shading conditions [3]. These types of functions are ideal for evaluating the performance of numerical optimization algorithms to find a global maximum [4,5]. Therefore, the data obtained can be used to evaluate the performance of maximum power point tracking controllers that use intelligent control techniques (such as fuzzy logic and neural networks) [6] and numerical optimization algorithms, in scenarios that simulate extreme conditions of irradiance and operating temperature [7,8].

When evaluating multimodal functions with optimization algorithms, individual contributions of PV modules can be detected [1]. For this reason, the algorithm is not able to optimize the entire function. In that sense, the data obtained with an artificial neural network are presented in order to have in a single function all the contributions of the PV modules.

For the training of the neural network, a normalization of the data was performed at an interval of [−1,1]. Once the network was trained, the inverse of normalization was implemented to return the data to the original scale. For this, a feedforward network with a hidden layer, 25 neurons and an output layer with a single neuron was used. A hyperbolic tangent sigmoid transfer function was used for each neuron.

The data obtained with the neural networks to approximate the multimodal functions can be used to evaluate the performance of different system identification techniques [9], using tools such as the System Identification Toolbox for Matlab, which allows representing the dynamics of nonlinear systems as presented in this work.
