# Supplementary material for: Data from multimodal functions based on an array of photovoltaic modules and an approximation with artificial neural networks as a scenario for testing optimization algorithms
Source: Data Brief. 2019 Oct 16;27:104669. doi: 10.1016/j.dib.2019.104669 (PMC6833458; doi:10.1016/j.dib.2019.104669)
Supplement: Multimedia component 2 [file mmc2.pdf]

```c
#include<stdio.h>
#include<stdlib.h>
#include<math.h>

void ortiz_init(float p, float s, float Ei, float Ein, float Tn, float Isc, float
Voc, float T, float TCv, float TCi, float *Vx, float *Ix)
{

	float Vmax=0.0;
	float Vmin=0.0;

	Vmax=Voc*1.03;
	Vmin=Voc*0.5;

	*Ix = p*(Ei/Ein)*(Isc+(TCi*(T-Tn)));
	*Vx = s*(Ei/Ein)*TCv*(T-Tn)+(s*Vmax)-(s*(Vmax-Vmin))*exp((Ei/Ein)*(log((Vmax-
Voc)/(Vmax-Vmin))));
}

void ortiz_potencia(float Vx, float Ix, float Vi, float b, float *Iv, float *Pot)
{
	*Iv = (Ix/(1-(exp(-1/b))))*(1-(exp((Vi/(b*Vx))-(1/b))));
	*Pot = Vi*(*Iv);
}

int mayor(int x0, int x1, int x2, int x3, int x4, int *sw)
{

	int xm=0;

	if(x0>x1 && x0>x2 && x0>x3 && x0>x4)
	{
		xm=x0;
		*sw=0;
	}
	else if(x1>x0 && x1>x2 && x1>x3 && x1>x4)
	{
		xm=x1;
		*sw=1;
	}
	else if(x2>x0 && x2>x1 && x2>x3 && x2>x4)
	{
		xm=x2;
		*sw=2;
	}
	else if(x3>x0 && x3>x1 && x3>x2 && x3>x4)
	{
		xm=x3;
		*sw=3;
	}

	else if(x4>x0 && x4>x1 && x4>x2 && x4>x3)
	{
		xm=x4;
		*sw=4;
	}

	return xm;
}

int main(void)
{
```

```
int s=0;
int p=0;
float Tn=0.0;
float b=0.0;
float Isc=0.0;
float Voc=0.0;

float TCv=0.0;
float TCi=0.0;
float Ein=1000.0;
float step=0.0;
int i=0, sw=0;

int   finc0=0, finc1=0, finc2=0, finc3=0, finc4=0, fincP=0;
float Vx0=0.0, Vx1=0.0, Vx2=0.0, Vx3=0.0, Vx4=0.0;
float Ix0=0.0, Ix1=0.0, Ix2=0.0, Ix3=0.0, Ix4=0.0;
float Iv0=0.0, Iv1=0.0, Iv2=0.0, Iv3=0.0, Iv4=0.0;
float Vi0=0.0, Vi1=0.0, Vi2=0.0, Vi3=0.0, Vi4=0.0;
float Pot0=0.0, Pot1=0.0, Pot2=0.0, Pot3=0.0, Pot4=0.0, PotP=0.0;
float *vecP0, *vecP1, *vecP2, *vecP3, *vecP4;
float *vecVi0, *vecVi1, *vecVi2, *vecVi3, *vecVi4, *vecViP;

float Ei0=10.0, Ei1=100.0, Ei2=700.0, Ei3=100.0, Ei4=500.0;
float T0=5.0, T1=45.0, T2=150.0, T3=10.0, T4=150.0;

FILE *potencia0;
FILE *potencia1;
FILE *potencia2;
FILE *potencia3;
FILE *potencia4;

FILE *parcial;

s = 1;
p = 1;
Tn = 25;
b = 0.0684;
Isc = 3.71;
Voc = 21.40;
TCv = -0.1261;
TCi = 0.00418;
step=0.01;

ortiz_init(p,s,Ei0,Ein,Tn,Isc,Voc,T0,TCv,TCi,&Vx0,&Ix0);
ortiz_init(p,s,Ei1,Ein,Tn,Isc,Voc,T1,TCv,TCi,&Vx1,&Ix1);
ortiz_init(p,s,Ei2,Ein,Tn,Isc,Voc,T2,TCv,TCi,&Vx2,&Ix2);
ortiz_init(p,s,Ei3,Ein,Tn,Isc,Voc,T3,TCv,TCi,&Vx3,&Ix3);
ortiz_init(p,s,Ei4,Ein,Tn,Isc,Voc,T4,TCv,TCi,&Vx4,&Ix4);

finc0=(int)(Vx0/step);
finc1=(int)(Vx1/step);
finc2=(int)(Vx2/step);
finc3=(int)(Vx3/step);
finc4=(int)(Vx4/step);

vecP0 = (float *)malloc((int)finc0*sizeof(float));
vecP1 = (float *)malloc((int)finc1*sizeof(float));
vecP2 = (float *)malloc((int)finc2*sizeof(float));
vecP3 = (float *)malloc((int)finc3*sizeof(float));
vecP4 = (float *)malloc((int)finc4*sizeof(float));

vecVi0 = (float *)malloc((int)finc0*sizeof(float));
```

```
vecVi1 = (float *)malloc((int)finc1*sizeof(float));
vecVi2 = (float *)malloc((int)finc2*sizeof(float));
vecVi3 = (float *)malloc((int)finc3*sizeof(float));
vecVi4 = (float *)malloc((int)finc4*sizeof(float));

potencia0=fopen("potencia0.dat","w");
potencia1=fopen("potencia1.dat","w");
potencia2=fopen("potencia2.dat","w");
potencia3=fopen("potencia3.dat","w");
potencia4=fopen("potencia4.dat","w");

parcial=fopen("parcial.dat","w");

for(i=0;i<finc0;i++)
{
        Vi0=Vi0+step;
        vecVi0[i]=Vi0;
        ortiz_potencia(Vx0,Ix0,Vi0,b,&Iv0,&Pot0);
        fprintf(potencia0,"%f  %f\n",Vi0,Pot0);
        vecP0[i]=Pot0;
}

for(i=0;i<finc1;i++)
{
        Vi1=Vi1+step;
        vecVi1[i]=Vi1;
        ortiz_potencia(Vx1,Ix1,Vi1,b,&Iv1,&Pot1);
        fprintf(potencia1,"%f  %f\n",Vi1,Pot1);
        vecP1[i]=Pot1;
}

for(i=0;i<finc2;i++)
{
        Vi2=Vi2+step;
        vecVi2[i]=Vi2;
        ortiz_potencia(Vx2,Ix2,Vi2,b,&Iv2,&Pot2);
        fprintf(potencia2,"%f  %f\n",Vi2,Pot2);
        vecP2[i]=Pot2;
}

for(i=0;i<finc3;i++)
{
        Vi3=Vi3+step;
        vecVi3[i]=Vi3;
        ortiz_potencia(Vx3,Ix3,Vi3,b,&Iv3,&Pot3);
        fprintf(potencia3,"%f  %f\n",Vi3,Pot3);
        vecP3[i]=Pot3;
}

for(i=0;i<finc4;i++)
{
        Vi4=Vi4+step;
        vecVi4[i]=Vi4;
        ortiz_potencia(Vx4,Ix4,Vi4,b,&Iv4,&Pot4);
        fprintf(potencia4,"%f  %f\n",Vi4,Pot4);
        vecP4[i]=Pot4;
}

fincP=mayor(finc0,finc1,finc2,finc3,finc4,&sw);

vecViP = (float *)malloc((int)fincP*sizeof(float));
```

```
        switch(sw)
        {
                case 0:
                        for(i=0;i<fincP;i++)
                        {
                                vecViP[i]=vecVi0[i];
                        }
                        break;
                case 1:
                        for(i=0;i<fincP;i++)
                        {
                                vecViP[i]=vecVi1[i];
                        }
                        break;
                case 2:
                        for(i=0;i<fincP;i++)
                        {
                                vecViP[i]=vecVi2[i];
                        }
                        break;
                case 3:
                        for(i=0;i<fincP;i++)
                        {
                                vecViP[i]=vecVi3[i];
                        }
                        break;
                default:
                        for(i=0;i<fincP;i++)
                        {
                                vecViP[i]=vecVi4[i];
                        }
                        break;
        }

        for(i=0;i<fincP;i++)
        {
                PotP = vecP0[i] + vecP1[i] + vecP2[i] + vecP3[i] + vecP4[i];
                fprintf(parcial,"%f  %f\n",*(vecViP+i), PotP);
        }

        free(vecP0);
        free(vecP1);
        free(vecP2);
        free(vecP3);
        free(vecP4);

        free(vecVi0);
        free(vecVi1);
        free(vecVi2);
        free(vecVi3);
        free(vecVi4);

        free(vecViP);

        return 0;
}
```
